# Artificial Intelligence Mapping of Structure to Function in Glaucoma

**DOI:** 10.1167/tvst.9.2.19

**Published:** 2020-03-30

**Authors:** Eduardo B. Mariottoni, Shounak Datta, David Dov, Alessandro A. Jammal, Samuel I. Berchuck, Ivan M. Tavares, Lawrence Carin, Felipe A. Medeiros

**Affiliations:** 1 Vision, Imaging and Performance (VIP) Laboratory, Duke Eye Center, Duke University, Durham, NC, USA; 2 Department of Ophthalmology and Visual Sciences, Paulista School of Medicine, Universidade Federal de Sao Paulo, São Paulo, Brazil; 3 Department of Electrical and Computer Engineering, Duke University, Durham, NC, USA; 4 Department of Statistical Science and Forge, Duke University, Durham, NC, USA

**Keywords:** glaucoma, optical coherence tomography, artificial intelligence, deep learning, machine learning

## Abstract

**Purpose:**

To develop an artificial intelligence (AI)–based structure-function (SF) map relating retinal nerve fiber layer (RNFL) damage on spectral domain optical coherence tomography (SDOCT) to functional loss on standard automated perimetry (SAP).

**Methods:**

The study included 26,499 pairs of SAP and SDOCT from 15,173 eyes of 8878 patients with glaucoma or suspected of having the disease extracted from the Duke Glaucoma Registry. The data set was randomly divided at the patient level in training and test sets. A convolutional neural network (CNN) was initially trained and validated to predict the 52 sensitivity threshold points of the 24-2 SAP from the 768 RNFL thickness points of the SDOCT peripapillary scan. Simulated localized RNFL defects of varied locations and depths were created by modifying the normal average peripapillary RNFL profile. The simulated profiles were then fed to the previously trained CNN, and the topographic SF relationships between structural defects and SAP functional losses were investigated.

**Results:**

The CNN predictions had an average correlation coefficient of 0.60 (*P* < 0.001) with the measured values from SAP and a mean absolute error of 4.25 dB. Simulated RNFL defects led to well-defined arcuate or paracentral visual field losses in the opposite hemifield, which varied according to the location and depth of the simulations.

**Conclusions:**

A CNN was capable of predicting SAP sensitivity thresholds from SDOCT RNFL thickness measurements and generate an SF map from simulated defects.

**Translational Relevance:**

AI-based SF map improves the understanding of how SDOCT losses translate into detectable SAP damage.

## Introduction

Glaucoma is a progressive optic neuropathy in which structural damage to the optic nerve and retinal nerve fiber layer (RNFL)^1^ is often accompanied by characteristic patterns of visual field defects. Understanding the consequences of structural damage on visual function (i.e., the structure-function [SF] relationship) is essential to allow proper diagnosis of glaucoma and discrimination from other diseases that may affect the visual system, as well as to provide prognostic information.

Previous attempts to map the SF relationship in glaucoma have mostly relied on oversimplified assumptions or on relatively small samples. For example, in a study by Garway-Heath et al.,^2^ localized RNFL defects seen on red-free photographs were mapped to the location of points on standard automated perimetry (SAP) in 63 patients. The authors then built a correspondence map of structure and function, which has been widely used and validated in clinical practice. The Garway-Heath map relied on subjective detection of visible RNFL defects on photographs. However, experimental and clinical studies have shown that such defects only appear when a substantial proportion of the RNFL has been lost.^3^^,^^4^ In addition, due to the limited number of eyes available in the study and the difficulties in visualizing RNFL defects in certain regions around the optic nerve, certain topographic relationships may have been left underappreciated.

Imaging of the RNFL with spectral-domain optical coherence tomography (SDOCT) is able to provide reproducible and quantitative assessment of the RNFL to a much greater degree than what is possible by assessing red-free RNFL photographs. Many previous studies have assessed the relationship between SDOCT and SAP.^5^^–^^7^ However, given the large amount of data provided by these tests, it can be difficult to apply conventional statistical tools to adequately model relevant SF relationships. Recently, neural networks and other artificial intelligence (AI) algorithms have been shown to successfully model complex, nonlinear relationships in data from diverse medical fields.^8^^–^^12^ In particular, convolutional neural networks (CNNs) are able to take advantage of spatial information to identify underlying relationships that may not be easily discerned by conventional methods. A few studies have attempted to use AI algorithms to predict visual field results from SDOCT measurements, with good results.^13^^–^^16^ In one study, Guo et al.^13^ showed that SAP sensitivity thresholds could be reasonably predicted from RNFL and ganglion cell and inner plexiform layer thicknesses. Using SDOCT volume scans of optic nerve head and macula, Maetschke et al.^14^ were able to predict visual field global metrics, such as mean deviation and visual field index. These studies were in general concerned about how well the SAP sensitivity thresholds, predefined sectors, or global metrics could be approximated by SDOCT data but did not evaluate the topographic mapping and spatial relationship between structural and functional damage, which is by itself another important issue.

We hypothesized that once an AI model is trained to predict SAP sensitivity thresholds, one could obtain topographical information of the SF relationship by simulating RNFL defects of varying characteristics and observing their impact on SAP results. This would allow a more complete investigation of the effects of structural damage seen on SDOCT on visual function as measured by SAP. To that effect, in this study, we developed and validated a CNN that predicts SAP sensitivity thresholds from peripapillary SDOCT RNFL thickness measurements in a large clinical cohort of patients with glaucoma and suspected of disease. We then applied an innovative simulation approach that allowed us to characterize the impact of the extension and depth of SDOCT RNFL defects on SAP results, providing important information on the SF relationship in glaucoma.

## Methods

This was a retrospective study that used cross-sectional data from the Duke Glaucoma Repository, a database of electronic medical and research records at the Vision, Imaging, and Performance Laboratory at Duke University. The institutional review board approved this study, and a waiver of informed consent was granted due to the retrospective nature of this work. All methods adhered to the tenets of the Declaration of Helsinki for research involving human participants, and the study was conducted in accordance with regulations of the Health Insurance Portability and Accountability Act.

Patients had a diagnosis of glaucoma or were suspected of having glaucoma and completed at least one SDOCT and one SAP visit within 180 days of each other. Patients who had procedures (e.g., panretinal photocoagulation) or other diseases that could impact the RNFL thickness measurements from SDOCT or visual field (e.g., retinal detachment, optic neuritis, proliferative diabetic retinopathy, intraocular tumors, vascular occlusions) were excluded. Patients younger than 18 years were also excluded. An additional group of 1827 SDOCT tests from 463 eyes of 235 healthy individuals was included in the study to represent the normal RNFL thickness profile in the structure-function map.

The visual field tests were performed using SAP with the 24-2 Swedish Interactive Threshold Algorithm (Carl Zeiss Meditec, Inc., Dublin, CA, USA) protocol. Unreliable tests with more than 33% fixation losses or more than 15% false-positive errors were excluded. After excluding the two points around the blind spot, the 52 sensitivity thresholds for each test were extracted.

RNFL thickness was collected from peripapillary RNFL scans, acquired using the Spectralis SDOCT (version 5.4.7.0; Heidelberg Engineering, GmbH, Dossenheim, Germany). The software provides measurements of the RNFL at 768 evenly spaced points in a circle of 3.45 mm of diameter positioned around the center of Bruch's membrane opening. Tests with a quality score lower than 15 were excluded according to manufacturer recommendations.

### CNN Algorithm

A CNN was developed to predict the 52 SAP sensitivity threshold values from the 768 peripapillary RNFL thickness points in SDOCT. Development and training of the algorithm were performed in Python, within Keras.^17^ The data set comprised a total of 26,499 pairs of SAP and SDOCT tests from 15,173 eyes of 8878 patients taken within an interval of 180 days. Of those, a set of 4494 pairs, from 1873 eyes of 1017 patients who were not included in the development of the CNN, was reserved to test the performance of the algorithm. The remaining 22,005 pairs were used for training and fine-tuning/validation of the model. Importantly, all randomizations between sets were performed at the patient level, so that no patient was present in more than one set.

The CNN had two hidden layers with one-dimension convolutional filters, which preserve the spatial relationship of the input data. The convolutional layers had 32 and 64 kernels of size 3. They were followed by two fully connected layers, with 54 and 52 nodes each. A nonlinear activation function (rectified linear unit) was applied after each hidden layer. The last fully connected layer, of size 52 (for each sensitivity threshold value in the 24-2 SAP), had an output with a linear activation. The algorithm was trained with stochastic gradient descent, optimized by the Adam algorithm.^18^ The initial learning rate was 1 × 10^–^^3^ and the algorithm was trained for 100 epochs, in which the weights of the epoch with the lowest mean squared error in the validation set were recorded. Importantly, no clinical assumptions (e.g., inferior hemiretina being related to the superior hemifield) were added to the model. Therefore, the topographic relationship was exclusively gleamed from the data, without using any previous clinical knowledge.

The performance of the CNN was evaluated through Pearson's correlation coefficients between predictions and the measured sensitivities, as well as through the mean absolute error (MAE) of the predictions in the test set. A simple ordinary least squares linear regression model, using the same inputs and outputs as the CNN, was built for comparative purposes.

To summarize the performance of the models by regions of the SAP, we averaged the measured sensitivity thresholds and the predictions by Garway-Heath sectors.^2^ The sector averages were then used to calculate sectoral MAE and correlation coefficients from both models.

### Simulations and Structure-Function Map

**Figure 1.**
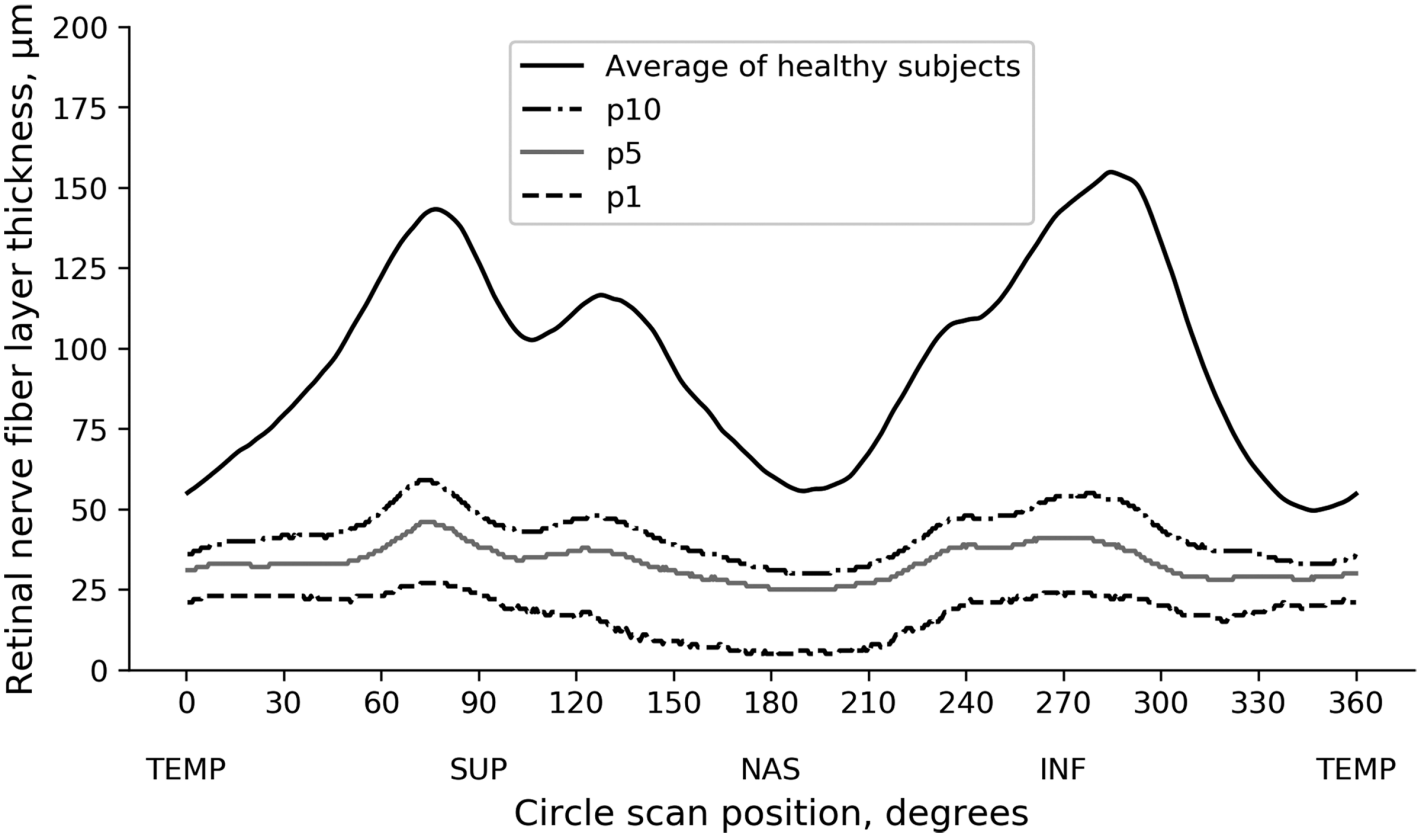
Retinal nerve fiber layer profile of average of healthy participants and pointwise percentiles from patients with glaucoma and glaucoma suspects.

Once the CNN was trained and validated, we performed a series of simulations to investigate the impact of structural RNFL damage on visual field loss. The simulations consisted of modifying a normal average peripapillary RNFL profile (i.e., the normal average of the 768 points) (Fig. 1) by simulating defects of varying locations and depths. The 768-point RNFL profile was initially divided in 12 evenly spaced 30° sectors (corresponding to clock-hour sectors). For each sector, we then simulated defects with depths corresponding to the 10th, 5th, and 1st percentiles of RNFL thickness derived from the glaucoma population (Fig. 1). Each simulated RNFL profile was then input into the previously trained CNN, and we observed the corresponding prediction of SAP sensitivity threshold values. This allowed us to obtain precise information on the functional effect of a specific structural loss.

**Figure 2.**
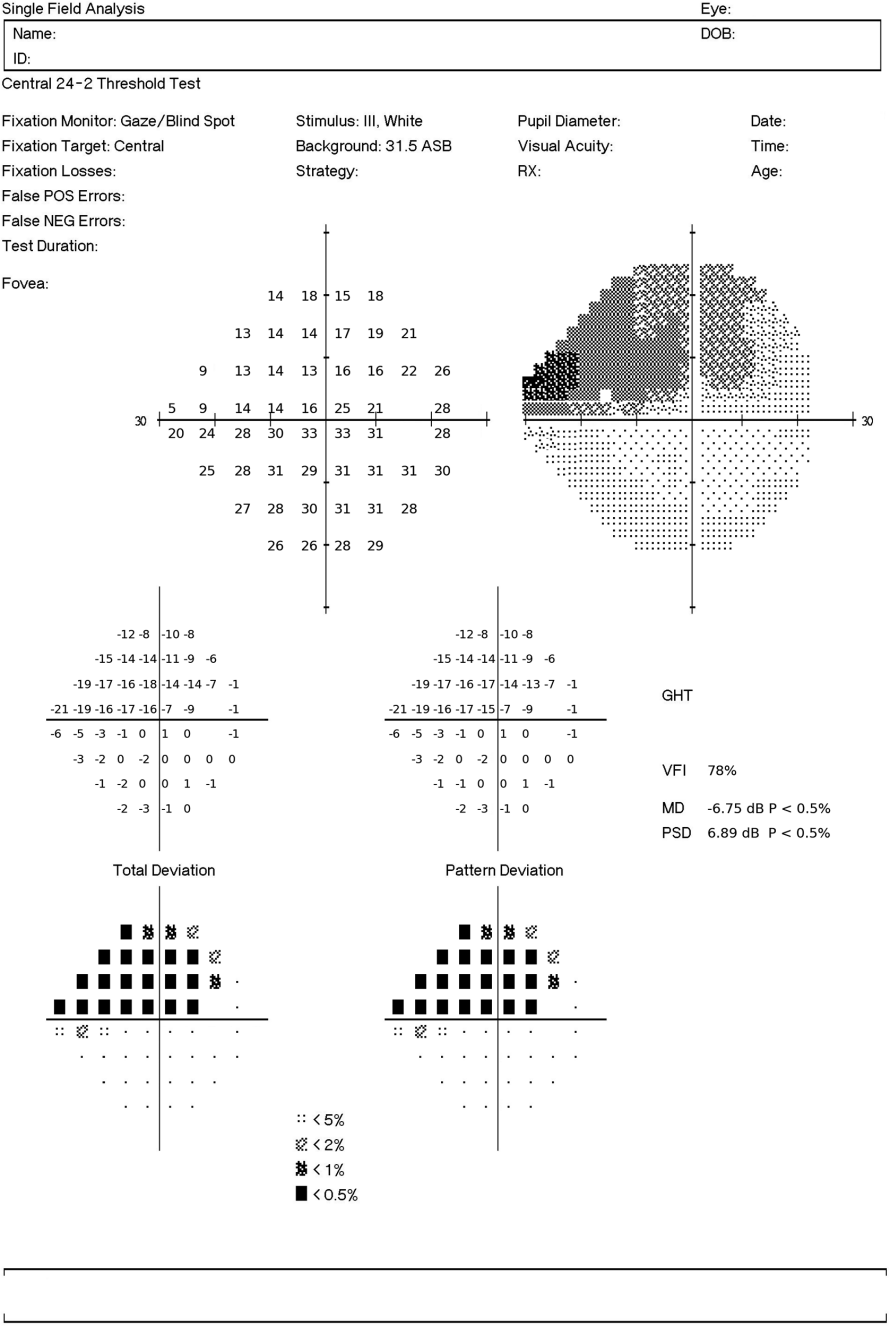
Example of the SAP report generated from CNN predictions of SAP sensitivity thresholds from optical coherence tomography RNFL thickness data. The RNFL profile used as input for the CNN had a simulated defect in the temporal inferior region (270°–300°) resulting in a superior visual field defect.

To enable the visualization of the visual field defect, we generated a report similar to the original printout of the SAP. The predictions from the CNN were used as sensitivity thresholds, and the total deviations were the difference between predictions and the age-corrected sensitivity thresholds based on healthy participants. The pattern deviations were the deviations from the seventh best value among the total deviation values. The probability plots were derived from percentiles for each point, compared with healthy participants. Figure 2 illustrates the visual field report generated from CNN predictions from a RNFL profile with a simulated defect on the temporal inferior region (270°–300°) resulting in a superior visual field defect.

### Statistical Analysis

Pearson's correlation coefficients between predictions and measured values were compared with a test of equality of correlations,^19^ while the absolute errors were compared with a random-effects mixed model,^20^ accounting for the presence of multiple tests from the same participant. A bootstrap resampling procedure was used to estimate 95% confidence intervals (CIs) for MAE and correlation coefficients.^21^ All statistical analyses were performed using Stata (version 15; StataCorp LP, College Station, TX, USA). The α level (type I error) was set at 0.05.

## Results

The data set comprised 26,499 pairs of SAP and SDOCT from 15,173 eyes of 8878 participants. The demographic and clinical information of the participants in the study are presented in Table 1. The demographic and clinical information of the healthy individuals whose SDOCT tests were used to derive the normal RNFL profile are available in Supplementary Table S1.

**Table 1.** Demographic and Clinical Information of the Participants on the Study

Characteristic	Training + Validation Set	Test Set
Number of SAP-SDOCT pairs	22,005	4494
Number of eyes	13,300	1873
Number of participants	7861	1017
Age at testing date, y	63.3 (14.6)	63.2 (14.3)
Sex, *n* (%)		
Female	4274 (54.4)	564 (55.5)
Race, *n* (%)		
Caucasian	4436 (56.4)	595 (58.5)
African American	2518 (32.0)	302 (29.7)
Asian	290 (3.7)	50 (4.9)
Hispanic	115 (1.5)	14 (1.4)
Other races	502 (6.4)	56 (5.5)
Diagnosis by eye, *n* (%)		
Glaucoma suspect	6313 (47.5)	821 (43.8)
Primary open-angle glaucoma	3916 (29.4)	611 (32.6)
Other glaucoma	3071 (23.1)	441 (23.6)
SAP mean deviation, dB	–5.10 (6.8)	–4.51 (6.0)
SAP pattern standard deviation, dB	4.01 (3.4)	3.87 (3.3)
SDOCT RNFL thickness, µm	78.3 (18.3)	77.5 (17.7)

Data presented as mean (standard deviation) unless otherwise noted.

**Figure 3.**
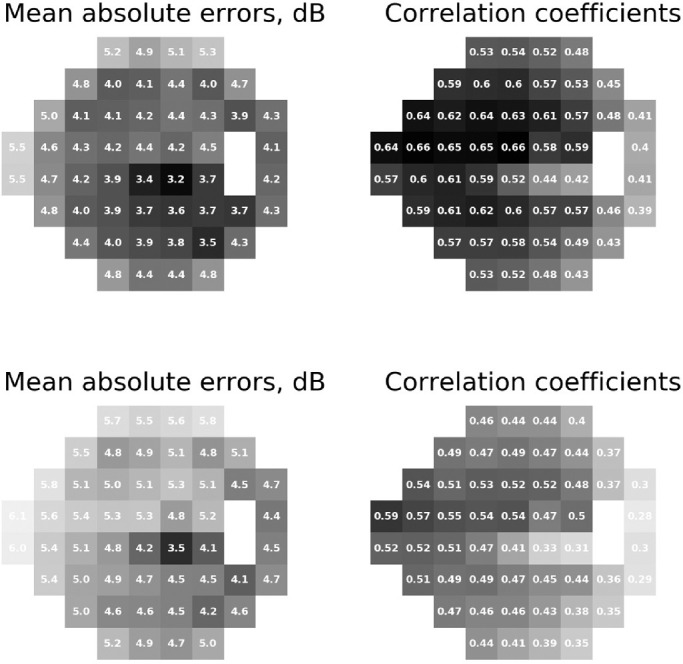
MAEs and correlation coefficients between the predictions from the convolutional neural network (*top*) and linear regression (*bottom*) and the measured sensitivity thresholds for each of the 52 locations tested by standard automated perimetry. The number inside each square represents the value for that specific location and the grayscale (*darker colors* represent lower MAEs and higher correlations with the measured values; *lighter colors* represent higher MAEs and lower correlation coefficients) illustrate the performance.

The test set comprised 4494 pairs of SAP and SDOCT from 1873 eyes of 1017 participants. Pearson's correlation coefficients between predicted and observed sensitivity threshold values for the 52 SAP locations ranged from 0.39 to 0.66, with an average *r* = 0.60 (*P* < 0.001; 95% CI, 0.58–0.63). The MAE error for each test location ranged from 3.15 to 5.53 dB, with an average MAE of 4.25 dB (95% CI, 4.06–4.44). The correlation coefficient between measured values and predictions from the linear regression model, developed for comparison purposes, ranged from 0.28 to 0.59, with an average *r* = 0.52 (*P* < 0.001; 95% CI, 0.49–0.55) and the MAE ranged from 3.54 to 6.08 dB, with an average of 4.96 dB (95% CI, 4.77–5.14 dB). The CNN performed better than the linear model by both correlation coefficients (*P* < 0.001) and MAE (*P* < 0.001). Figure 3 shows MAE and correlation coefficients of both models for each location of SAP. Table 2 presents the sectoral MAE and correlation coefficients of each model, according to Garway-Heath sectors.^2^

**Table 2.** Performance of the Convolutional Neural Network and the Linear Model Summarized by Garway-Heath Sectors

	CNN		Linear Regression	
Characteristic	MAE, dB (95% CI)	Pearson's *r* (95% CI)	MAE, dB (95% CI)	Pearson's *r* (95% CI)
Central	3.22 (2.03–3.42)	0.61 (0.56–0.65)	3.80 (3.61–3.99)	0.50 (0.46–0.54)
Temporal	3.77 (3.54–4.00)	0.44 (0.38–0.50)	4.16 (3.92–4.39)	0.32 (0.27–0.37)
Inferior	3.65 (3.44–3.86)	0.59 (0.55–0.64)	4.18 (3.97–4.40)	0.49 (0.44–0.53)
Inferior nasal	3.32 (3.11–3.53)	0.65 (0.62–0.69)	4.24 (4.03–4.46)	0.53 (0.49–0.56)
Superior	4.25 (4.03–4.46)	0.57 (0.52–0.62)	4.81 (4.58–5.03)	0.47 (0.42–0.51)
Superior nasal	3.68 (3.49–3.88)	0.68 (0.65–0.73)	4.56 (4.36–4.77)	0.58 (0.58–0.62)

**Figure 4.**
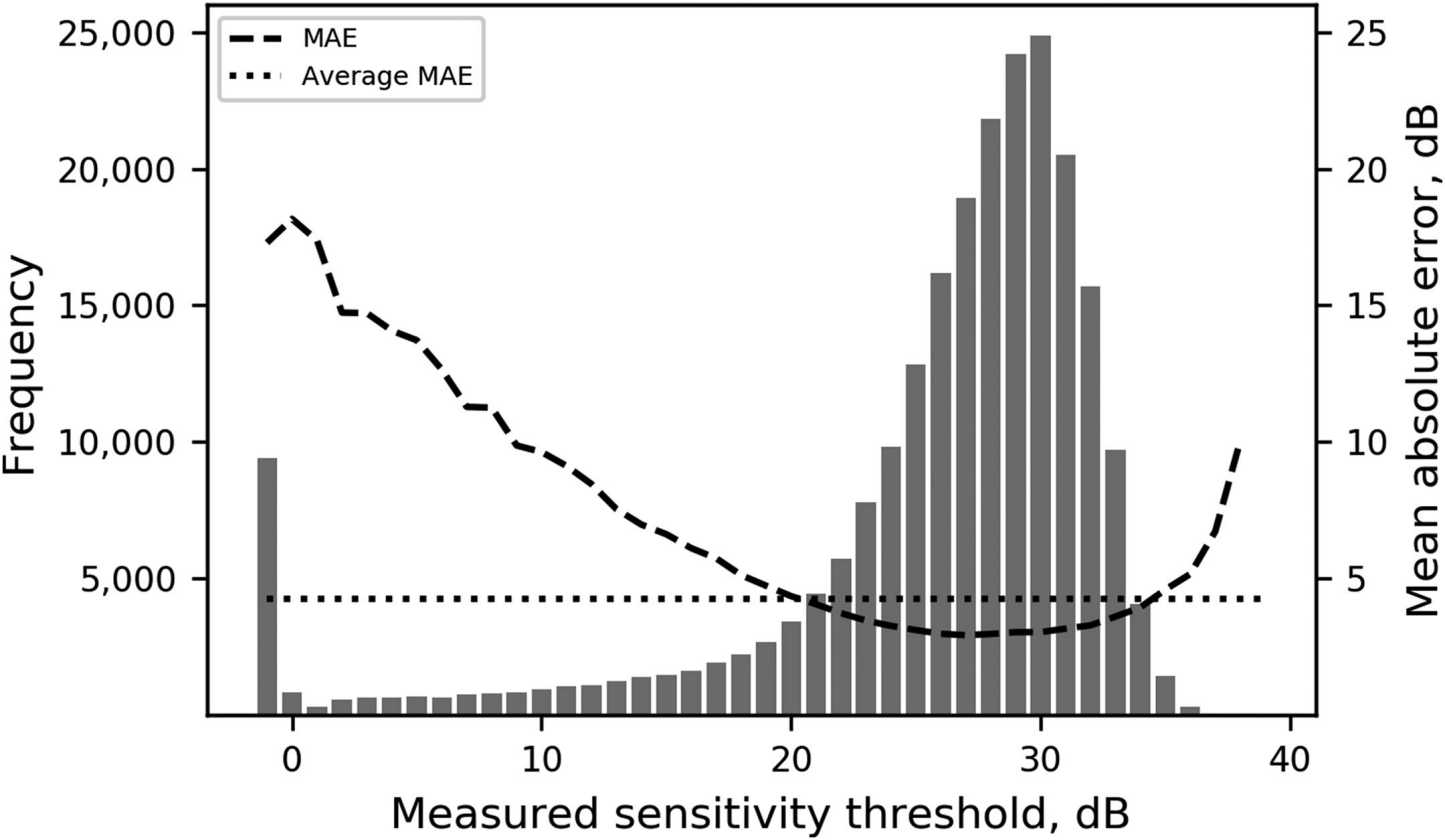
Histogram representing the frequency of sensitivity threshold values in the test set, measured by SAP. The *dashed line* represents the MAE of the predictions, grouped by bins of actual measured sensitivity threshold. The *dotted horizontal line* represents the average MAE of the model.

The performance of the CNN was better for sensitivity threshold values between 20 and 35 dB, which were the most frequent values in our sample, as Figure 4 illustrates. As a consequence, the absolute error of the CNN had higher correlation coefficients with SAP Mean deviation (MD) (*r* = –0.751, *P* < 0.001), SAP Pattern standard deviation (PSD) (*r* = 0.588, *P* < 0.001), and global RNFL thickness (*r* = –0.391, *P* < 0.001). Other factors such as age and SDOCT quality score had low correlation with the absolute error (Supplementary Table S2).

Supplementary Figure S1 illustrates an example where the CNN was able to predict the SAP accurately from the RNFL thickness measurements. Supplementary Figure S2 illustrates a case in which the CNN predictions had a high absolute error when compared to the measured SAP sensitivities.

### Simulation of RNFL Defects and Structure-Function Map

**Figure 5.**
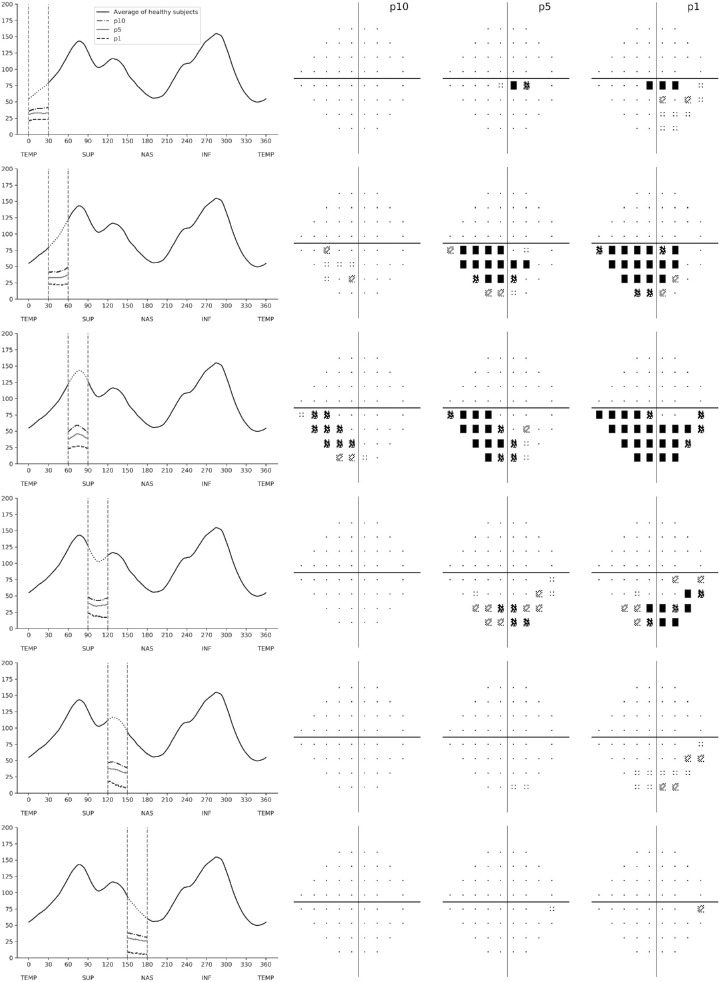
Patterns of visual field loss predicted from the convolutional neural network when simulating RNFL defects in the superior hemiretina. The RNFL profile is shown on the *left*, with *dashed vertical lines* showing the location of each simulated RNFL defect. For each simulated defect in a particular location, there were three simulated depths representing the 10th (p10), 5th (p5), and 1st (p1) percentiles. The corresponding predicted standard automated perimetry pattern deviation plots are shown on the *right*.

**Figure 6.**
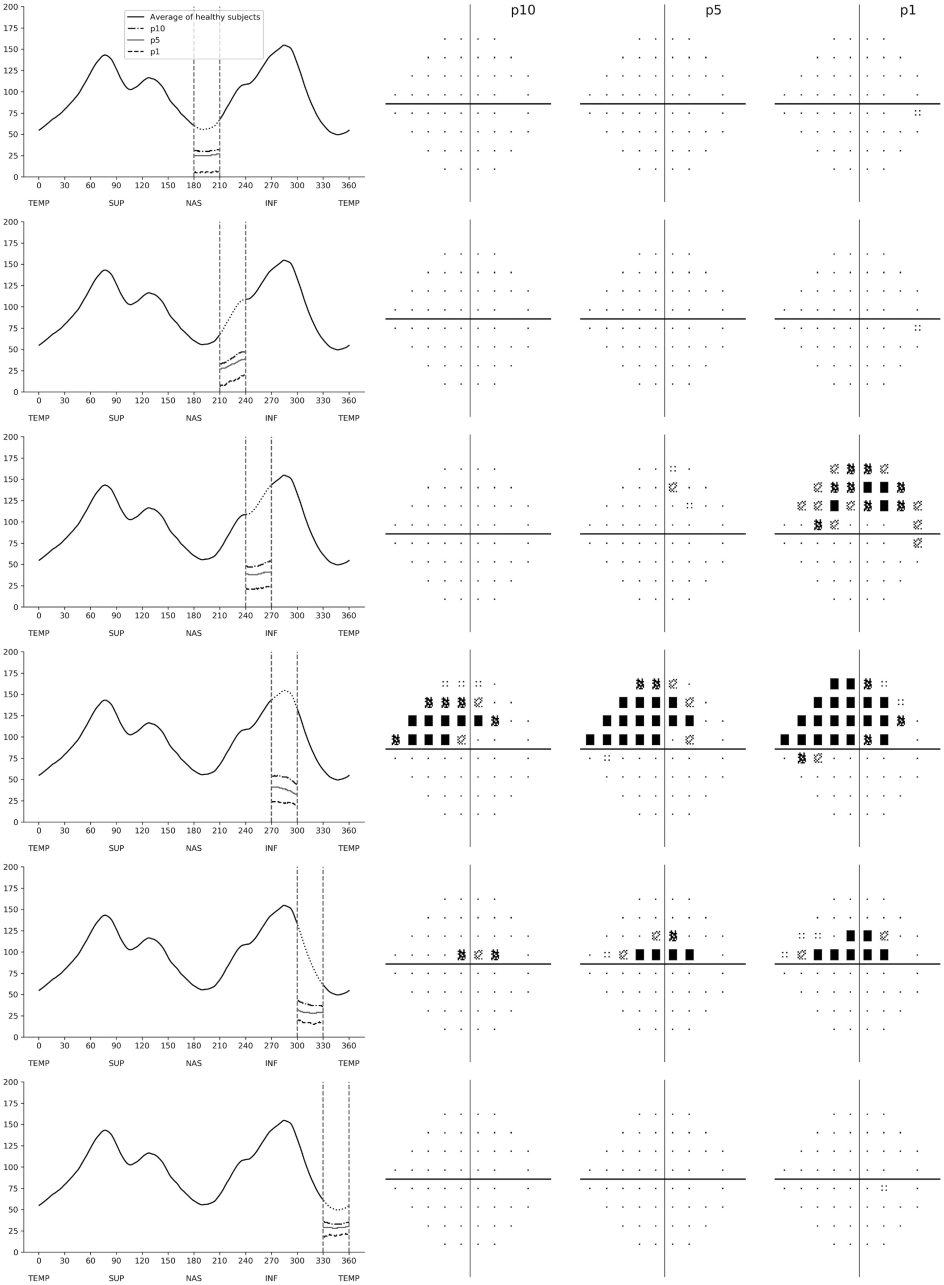
Patterns of visual field loss predicted from the convolutional neural network when simulating RNFL defects in the inferior hemiretina. The RNFL profile is shown on the *left*, with *dashed vertical lines* showing the location of the simulated RNFL defect. For each simulated defect in a particular location, there were three simulated depths representing the 10th (p10), 5th (p5), and 1st (p1) percentiles. The corresponding predicted standard automated perimetry pattern deviation plots are shown on the *right*.

Figures 5 and 6 show SAP predicted pattern deviation plots for the different simulated RNFL defects. The RNFL thickness profiles are represented in a temporal-superior-nasal-inferior-temporal fashion, with dashed vertical lines delineating the location of the sector where the RNFL defect was simulated. Figure 5 shows predictions from simulated defects in the superior RNFL sectors, going from the temporal to nasal regions. It can be seen that the simulated defects generated corresponding inferior losses in the visual field, as it would be expected. The pattern of visual field loss corresponded to the location and depth of the defects. Defects in the superior temporal sectors, such as from 30° to 60° and 60° to 90°, produced inferior arcuate visual field defects that were more extensive as the simulated RNFL defects got deeper. The superior temporal defect extending from 0° to 30° generated an inferior paracentral defect in the visual field. Interestingly, the inferior paracentral defect was only evident with defect depths in the 5th and 1st percentiles but not with a shallower defect at the 10th percentile. Also interesting, as the location of the simulated defects moved nasally, such as in the defects from 90° to 120° and 120° to 150°, the arcuate defect in the visual field became more peripheral. Finally, for the RNFL defect extending nasally from 150° to 180°, only a small single abnormal point was predicted in the temporal visual field.

The general pattern for defects simulated in the inferior RNFL region was very similar to those for the superior hemiretina (Fig. 6). As expected, simulated inferior localized RNFL defects generated corresponding superior visual field losses. For RNFL defects localized in the nasal most regions, such as from 180° to 210° and 210° to 240°, the visual fields were essentially normal, with just a few points abnormal in the temporal visual field. In contrast, inferior temporal RNFL defects generated inferior arcuate defects that got more pronounced the deeper the simulated RNFL defect was. The inferior RNFL defect extending from 300° to 330° generated a superior paracentral visual field defect. Interestingly, in contrast to the corresponding defect simulated in the superior RNFL, such defect was noticeable even at a relatively shallow depth of the 10th percentile. As expected, the paracentral defect got more pronounced the deeper the RNFL simulated loss was. Also of interest, and again in contrast with the superior RNFL, a simulated loss in the most temporal inferior sector did not result in a noticeable visual field defect.

To make qualitative comparisons with the SF map developed with the CNN, the linear regression model was also used to get predictions of the simulations. The linear model was not able to capture all the visual defects that the CNN did, particularly for defects simulated in the superior half of the RNFL (e.g., sectors between 0–30°, 90–120°), as illustrated in Supplementary Figure S3. All defects generated by the linear model were present in the SF map developed with the CNN.

**Figure 7.**
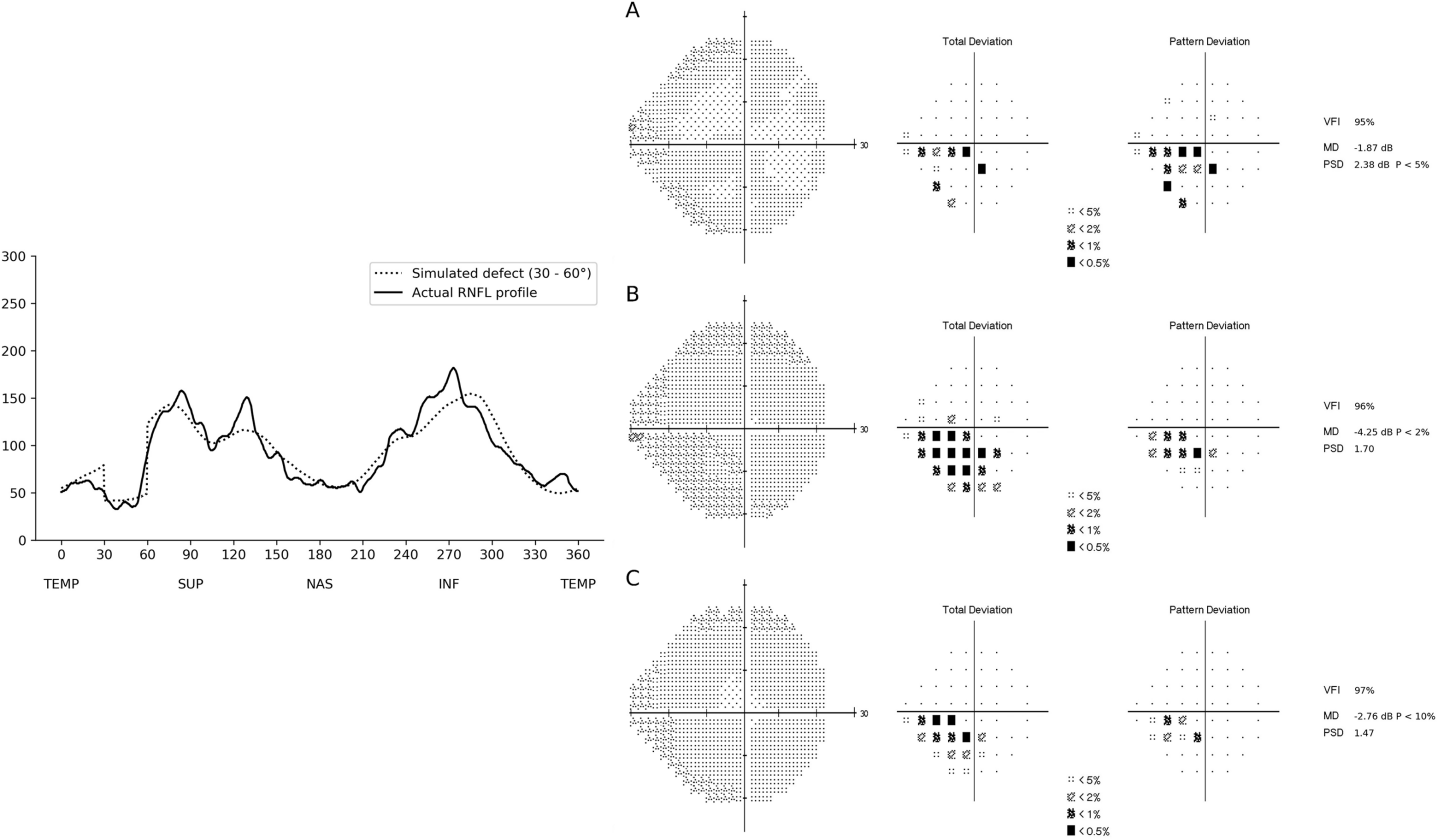
Example of a RNFL profile included in the test sample that closely resembled a RNFL profile with a localized defect in the temporal superior region of the RNFL (30°–60°). The actual RNFL thickness is represented on the *left* (*solid line*), while the *dotted line* represents the simulated RNFL thickness. The corresponding visual fields are represented on the *right*, where (A) represents the actual visual field of the individual, (B) represents the visual field predicted by the CNN using the actual RNFL thickness of the individual, and (C) represents the visual field predicted by the CNN using the simulated RNFL thickness profile. In this example, the localized defect in the temporal superior RNFL was associated with a nasal inferior defect on the visual field. The defects present on the predictions resembled the actual visual field in location and depth of defect.

**Figure 8.**
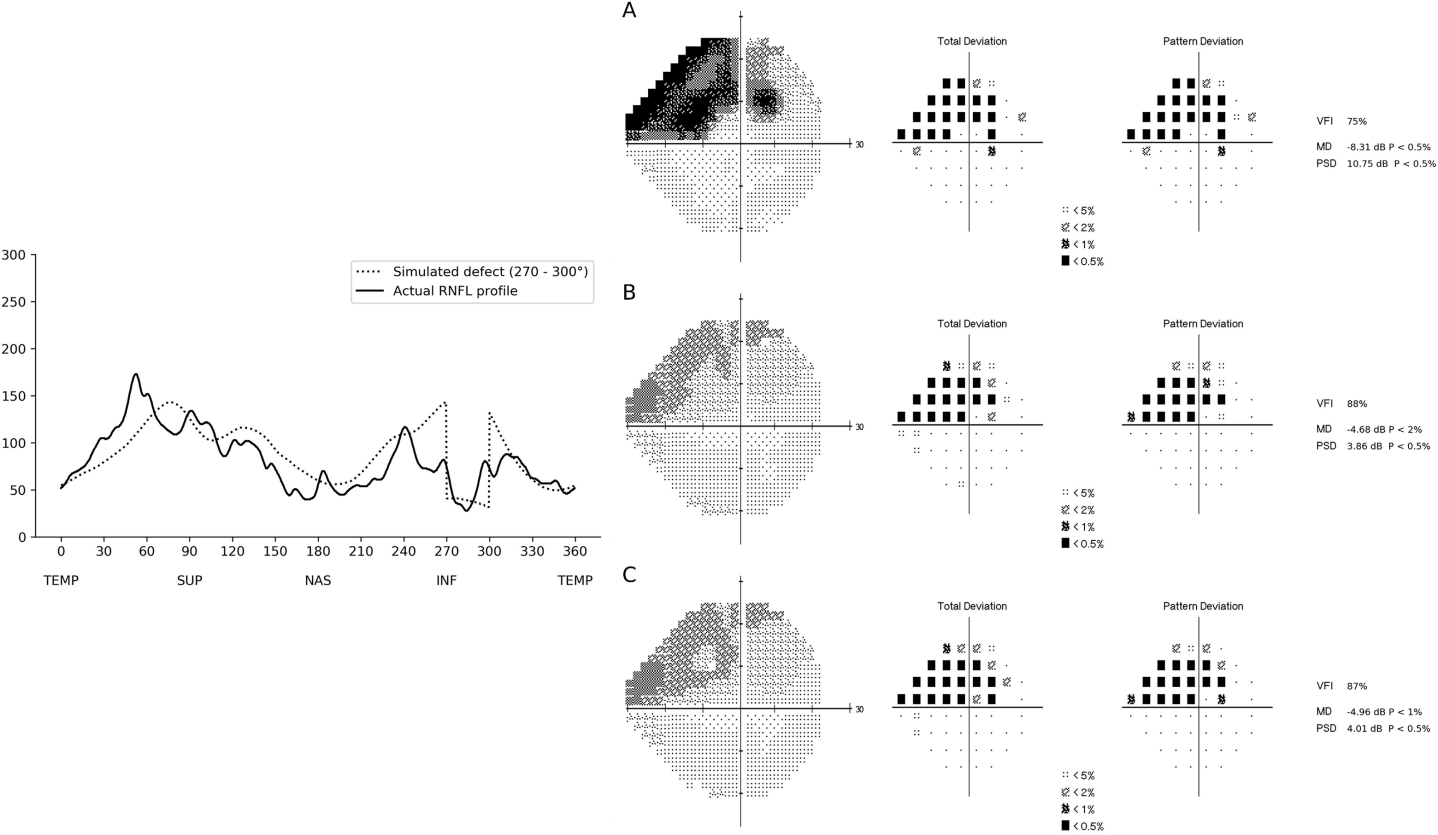
Example of a RNFL profile included in the test sample that closely resembled a RNFL profile with a localized defect in the temporal inferior region of the RNFL (270°–300°). The actual RNFL thickness is represented on the *left* (*solid line*), while the *dotted line* represents the simulated RNFL thickness. The corresponding visual fields are represented on the *right*, where (A) represents the actual visual field of the individual, (B) represents the visual field predicted by the CNN using the actual RNFL thickness of the individual, and (C) represents the visual field predicted by the CNN using the simulated RNFL thickness profile. In this example, the localized defect in the temporal inferior RNFL was associated with a superior arcuate defect on the visual field. The visual fields predicted by the CNN presented a defect with similar location and depth.

Although, by design, the simulated RNFL profiles were manufactured, they reflect a realistic spectrum of RNFL loss, as many localized defects from our training data resembled the simulations. Figures 7 and 8 show examples of localized defects in the temporal superior and temporal inferior regions, respectively.

## Discussion

In this study, we developed a CNN capable of predicting SAP sensitivity thresholds from SDOCT peripapillary RNFL thickness measurements, which were then subsequently used to produce SF maps relating functional losses to simulated RNFL defects. Such maps provided predictions of what visual field defects would look like for specific patterns of RNFL damage, according to the location and depth of RNFL defects. Such maps may improve our understanding of how SDOCT losses translate into detectable SAP defects and help to identify cases where SAP and optical coherence tomography (OCT) results are not compatible with the range of expected patterns, as it happens with unreliable tests or visual field losses due to other diseases, for example.

Exploring the visual field defects predicted by the CNN from these simulated defects, we were able to develop a structure-function map in patients with glaucoma. It was imperative that no previous knowledge about this relationship was introduced to the algorithm (e.g., using only the inferior RNFL to predict the superior SAP locations) so that all conclusions drawn from this map would be exclusively related to the data. Nonetheless, the patterns identified were similar to those expected from clinical experience. In agreement with previous maps, defects simulated on the temporal superior and temporal inferior regions of the RNFL resulted in visual field defects that were arcuate in shape in the inferior and superior hemifields, respectively. As the simulated defects moved nasally, the corresponding arcuate visual field defects became more peripheral, as expected from the arcuate RNFL anatomy. However, simulated RNFL defects on the most nasal side of the optic nerve did not trigger significant defects on the temporal visual field. The absence of a temporal wedge visual field defect may be related to a relative lack of SAP points covering the temporal region. In addition, given the rarity of such defects in patients with glaucoma, the relationship may not have been captured by the CNN.

When defects of the same depth were simulated on different regions of the RNFL, it resulted in visual field defects of varying magnitude. As an example, for sectors such as from 60° to 90° (temporal-superior) and 270° to 300° (temporal-inferior), which are also the most frequent regions of glaucomatous damage, a large arcuate defect in the visual field was present even when a relatively shallow defect (10th percentile) was simulated on the RNFL. In contrast, for sectors closer to the temporal disc, such as from 0° to 30° or from 330° to 360°, a RNFL defect in the 10th percentile did not result in appreciable visual field loss. This is in agreement with the work by Harwerth et al.,^22^ who investigated the magnitude of retinal ganglion cell loss required to produce a given visual field abnormality at different eccentricities. A paracentral loss at an eccentricity of 4.2°, for example, required four times the loss of retinal ganglion cells compared to an eccentricity of 24°. In addition, another explanation for the difficulty in producing paracentral defects may be the relatively sparse density of points of the 24-2 visual field test strategy in the macular area.

Investigating central visual field defects and macular glaucomatous damage, Hood et al.^23^^–^^25^ described the macular vulnerability zone (MVZ), a region that would approximately correspond to the sector between 300° and 330° in the inferior RNFL thickness profile. They associated defects on the MVZ to superior paracentral visual field defects. Accordingly, in our work, when defects were simulated in the sector from 300° to 330°, the CNN predicted a visual field with a corresponding paracentral superior defect, which supports their findings. Because of the relative anatomic positions of the optic nerve and macula, RNFL damage to the corresponding superior sector (30°–60°) actually tends to cause peripheral arcuate visual field defects inferiorly, rather than paracentral macular defects. As can be seen in Figure 7, a paracentral inferior visual field defect is actually caused by damage to the RNFL in the more temporal sector located from 0° to 30°.

Previous studies have also used machine learning to predict SAP thresholds from OCT measurements, with various approaches. Several attempts used previous clinical knowledge to improve their predictions.^13^^,^^15^^,^^16^^,^^26^^–^^32^ As an example, Guo et al.^13^ reported a root mean square error of 5.42 dB when predicting pointwise SAP sensitivity thresholds using RNFL and ganglion cell layer measurements as inputs. In their model, the use of selected regions presented better results than a naive approach that used all peripapillary RNFL. Other studies relied on different types of OCT inputs to get predictions of SAP.^7^^,^^33^^–^^37^ Christopher et al.^37^ achieved best performance when using en face images to predict SAP global metrics and sectoral averages. It should be noted, however, that our main purpose was to use the CNN to develop an SF map. For that reason, it was imperative that no previous clinical knowledge was included in the development of the CNN, so that all conclusions were gleamed exclusively from the data. It was also necessary that artificial RNFL defects could be simulated in the inputs, which was possible using the peripapillary RNFL thickness but would not be feasible for en face images or volumes.

This study has limitations. First, the ability to generate visual field defects from simulated RNFL defects is clearly dependent on the ability of the CNN to accurately learn to predict SAP thresholds. It is possible that patterns uncommon in the data, such as temporal wedges, were ignored by the network during the learning process. Second, the simulated defects used to develop the SF map can occasionally result in RNFL profiles that are not represented in our data set. Therefore, the simulations are not guaranteed to be reflective of clinical cases. In addition, as Figure 4 illustrates, the accuracy of the network to predict SAP sensitivity thresholds decreased considerably for thresholds below 15 dB. This may be a result of the fact that values below this level were much less common in our data, although attempts to oversample such abnormalities in our data did not improve the network performance (data not shown). Another reason for the decreased accuracy may be the large variability of individual SAP points once the sensitivity falls below 15 dB. In fact, previous studies have shown that once the sensitivity threshold reaches 15 dB, the variability may get as high as the measured threshold value itself.^38^^,^^39^ Finally, although the test set included images from a large population and was independent of the training/validation sample, it would be advantageous to evaluate the CNN using a test set from a new population.

In conclusion, we developed an AI-based mapping of structural OCT RNFL damage to SAP visual field loss in glaucoma. The derived map provides insights into the functional impact of RNFL defects of varying location and depth on OCT. Application of this algorithm may aid in the interpretation of OCT and SAP results in clinical practice and to assess the prognostic significance of RNFL defects in glaucoma.

## Supplementary Material

Supplement 1

Supplement 2

Supplement 3

Supplement 4

Supplement 5
