# Supplementary material for: Artificial Intelligence Mapping of Structure to Function in Glaucoma
Source: Transl Vis Sci Technol. 2020 Mar 30;9(2):19. doi: 10.1167/tvst.9.2.19 (PMC7395675; doi:10.1167/tvst.9.2.19)

Supplementary Figure S1. Example of a case where the convolutional neural network (CNN) was able to predict the visual field accurately using the retinal nerve fiber layer (RNFL) measurements. In the case illustrated, there is a large inferior temporal defect on the RNFL (left), that manifested on the visual field as a superior arcuate defect (A, right). The CNN predicted a visual field with a defect of similar shape and depth (B, right).

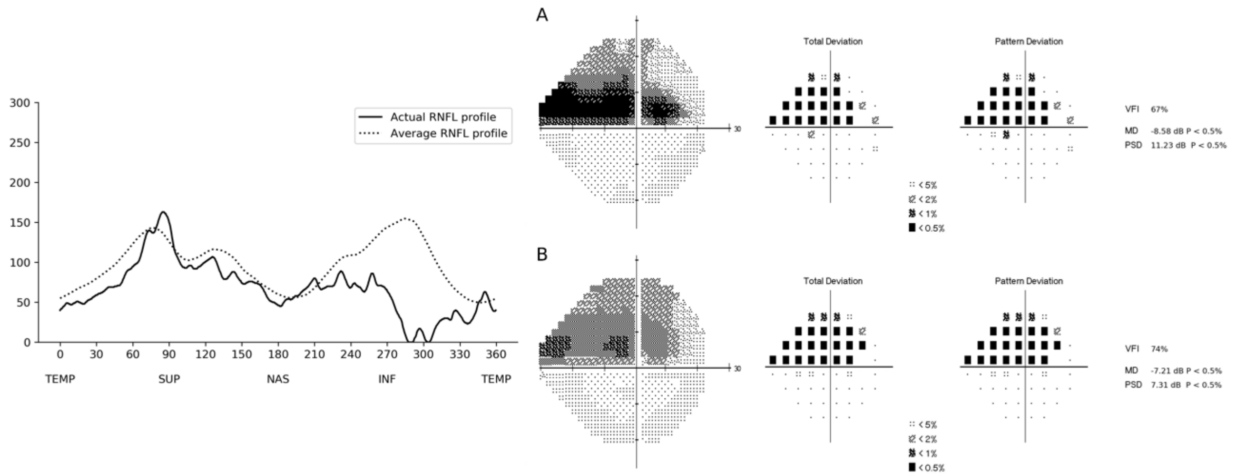

Supplement: Supplement 1 [file tvst-9-2-19_s001.pdf]
