# Supplementary material for: Artificial Intelligence Mapping of Structure to Function in Glaucoma
Source: Transl Vis Sci Technol. 2020 Mar 30;9(2):19. doi: 10.1167/tvst.9.2.19 (PMC7395675; doi:10.1167/tvst.9.2.19)

Supplementary Figure S2. Example of a case where the convolutional neural network (CNN) predictions obtained from retinal nerve fiber layer (RNFL) measurements had relatively high error compared to the actual visual field sensitivities. The actual visual field shows dense superior and inferior defects (A), whereas the predicted visual field (B) shows superior and inferior defects of similar shape, but considerably shallower than the original defects.

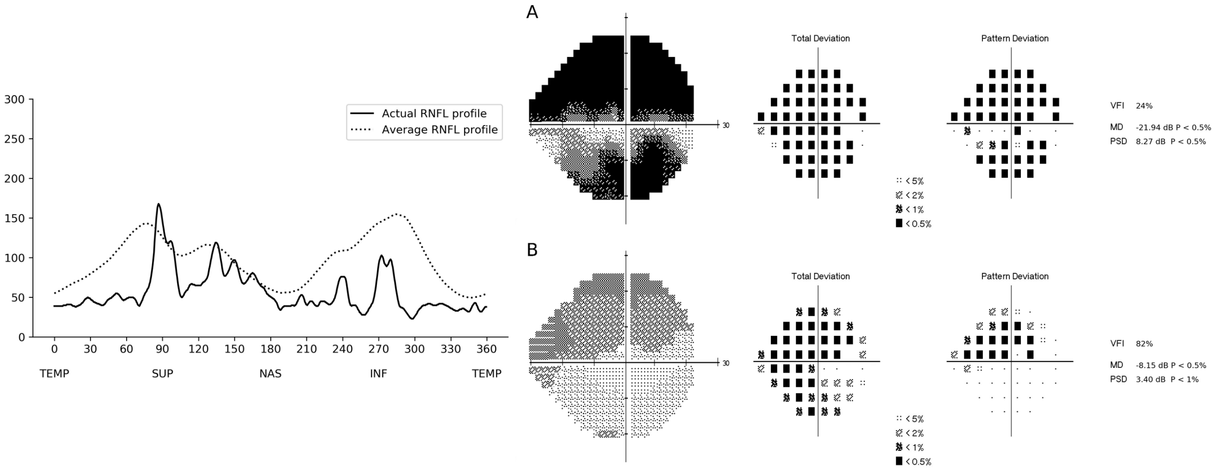

Supplement: Supplement 2 [file tvst-9-2-19_s002.pdf]
