# Supplementary material for: Artificial Intelligence Mapping of Structure to Function in Glaucoma
Source: Transl Vis Sci Technol. 2020 Mar 30;9(2):19. doi: 10.1167/tvst.9.2.19 (PMC7395675; doi:10.1167/tvst.9.2.19)

Supplementary Figure S3. Regions of the retinal nerve fiber layer (RNFL) where the convolutional neural networks (CNN; left) was able to capture a visual field loss and the linear regression model (right) failed. For a defect in the temporal region (0 – 30°), above the horizontal meridian, the CNN predicted a central defect in the inferior hemifield. For a defect simulated in the same sector, the linear model predicted a normal visual field, even with a deep (p1) defect. When a defect was simulated on the nasal superior region (90 – 120°, and 120 – 150°), the CNN predicted an incomplete arcuate defect in the inferior hemifield. Again, for the same sectors, the linear model predicted a visual field without any defects. For the other sectors, not represented in this figure, the pattern of visual field loss was similar for both models.

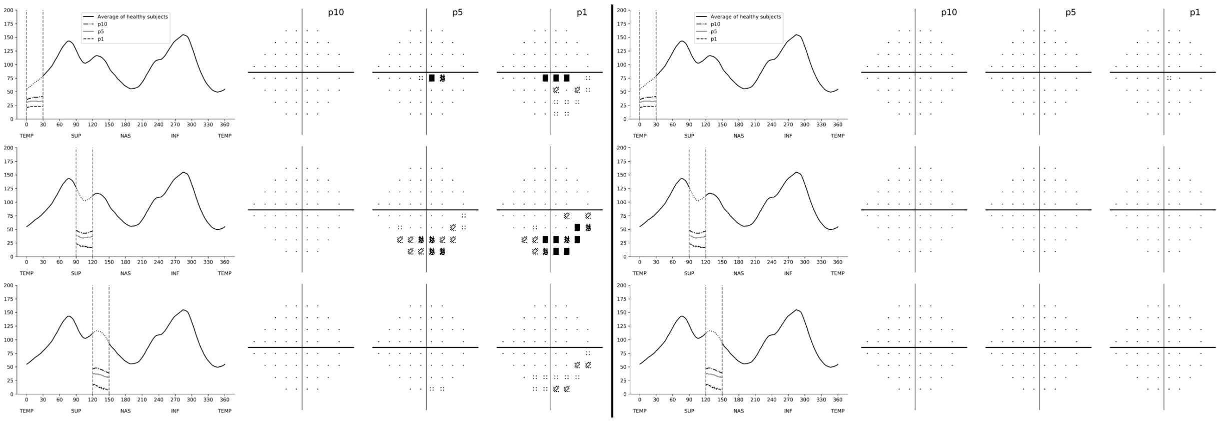

Supplement: Supplement 3 [file tvst-9-2-19_s003.pdf]
