# Supplementary material for: Artificial Intelligence Mapping of Structure to Function in Glaucoma
Source: Transl Vis Sci Technol. 2020 Mar 30;9(2):19. doi: 10.1167/tvst.9.2.19 (PMC7395675; doi:10.1167/tvst.9.2.19)
Supplement: Supplement 4 [file tvst-9-2-19_s004.pdf]

Supplementary Table S1. Demographical and Clinical information of healthy subjects included in the study.

	Healthy subjects
Number of eyes	463
Number of subjects	235
Mean age at testing date, years	56.6 (15.9)
Sex, (%)	
Female	139 (59.2)
Race, (%)	
Caucasian	105 (44.7)
African American	67 (28.5)
Asian	11 (4.7)
Hispanic	47 (20.0)
Other races	5 (2.1)
SDOCT RNFL thickness, μm	97.0 (10.8)

* Data presented as mean ± standard deviation, unless otherwise noted.
Abbreviations: SAP = standard automated perimetry; SDOCT = spectral domain optical coherence tomography; RNFL = retinal nerve fiber layer
