# Supplementary material for: Artificial Intelligence Mapping of Structure to Function in Glaucoma
Source: Transl Vis Sci Technol. 2020 Mar 30;9(2):19. doi: 10.1167/tvst.9.2.19 (PMC7395675; doi:10.1167/tvst.9.2.19)
Supplement: Supplement 5 [file tvst-9-2-19_s005.pdf]

Supplementary Table S2. Correlation coefficients between absolute error of the predictions and potential factors associated.

	Correlation coefficient (Pearson's r)	P value
**Age**	0.029	P = 0.049
**Interval of days between SDOCT and SAP**	0.054	P < 0.001
**SAP MD**	-0.751	P < 0.001
**SAP PSD**	0.588	P < 0.001
**RNFL thickness**		
**Global**	-0.391	P < 0.001
**Temporal**	-0.200	P < 0.001
**Temporal superior**	-0.382	P < 0.001
**Temporal inferior**	-0.380	P < 0.001
**Nasal**	-0.229	P < 0.001
**Nasal superior**	-0.258	P < 0.001
**Nasal inferior**	-0.282	P < 0.001
**SDOCT quality score**	-0.057	P < 0.001

Abbreviations: SDOCT = spectral-domain optical coherence tomography; SAP = standard automated perimetry; MD = mean deviation; PSD = pattern standard deviation; RNFL = retinal nerve fiber layer.
